# Supplementary material for: IGF2 Peptide-Based LYTACs for Targeted Degradation of Extracellular and Transmembrane Proteins
Source: Molecules. 2023 Nov 10;28(22):7519. doi: 10.3390/molecules28227519 (PMC10673611; doi:10.3390/molecules28227519)
Supplement: Supplementary file 1 [file molecules-28-07519-s001.zip › molecules-2706930-supplementary.pdf]

Supplementary Information

IGF2 Peptide-Based LYTACs for Targeted Degradation of Extracellular and Transmembrane Proteins

Michał Mikitiuk, Jan Barczyński, Przemysław Bielski, Marcelino Arciniega, Urszula Tyrcha, Aleksandra Hec, Andrea D. Lipińska, Michał Rychłowski, Tad A. Holak and Tomasz Sitar

List of Supplementary Figures

Supplementary Fig. S1 Determination of PD-L1-mCherry uptake by RL95-2 cells by fluorescence measurement.

Supplementary Fig. S2 Fluorescence microscopy images of RL95-2 cells treated with the C5M1A.

Supplementary Fig. S3 Fluorescence microscopy images of RL95-2 cells treated with the C5M1B.

Supplementary Fig. S4 Determination of cell surface PD-L1 levels by live cell flow cytometry of RL95-2 cells treated with C5M1A.

Supplementary Fig. S5 Results of PBMC cytotoxicity tests.

Supplementary Fig. S6 Cytotoxicity test of C5M1A.

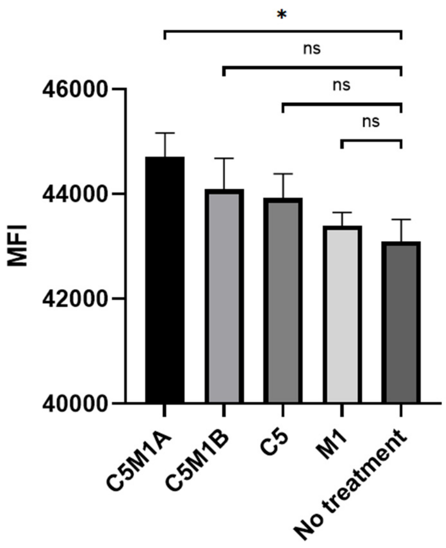

Supplementary Fig. S1 Determination of PD-L1-mCherry uptake by RL95-2 cells by fluorescence measurement. All compounds added at 100 nM, treated for 22 h. Data on all charts represent mean from 3 independent replicates as mean ± SD. The unpaired t-test was used to compare the means of each group against the untreated control. P value threshold of less than or equal to 0.05 was considered statistically significant. ns: $p > 0.05$; * $p \le 0.05$; ** $p \le 0.01$; *** $p \le 0.001$; **** $p \le 0.0001$.

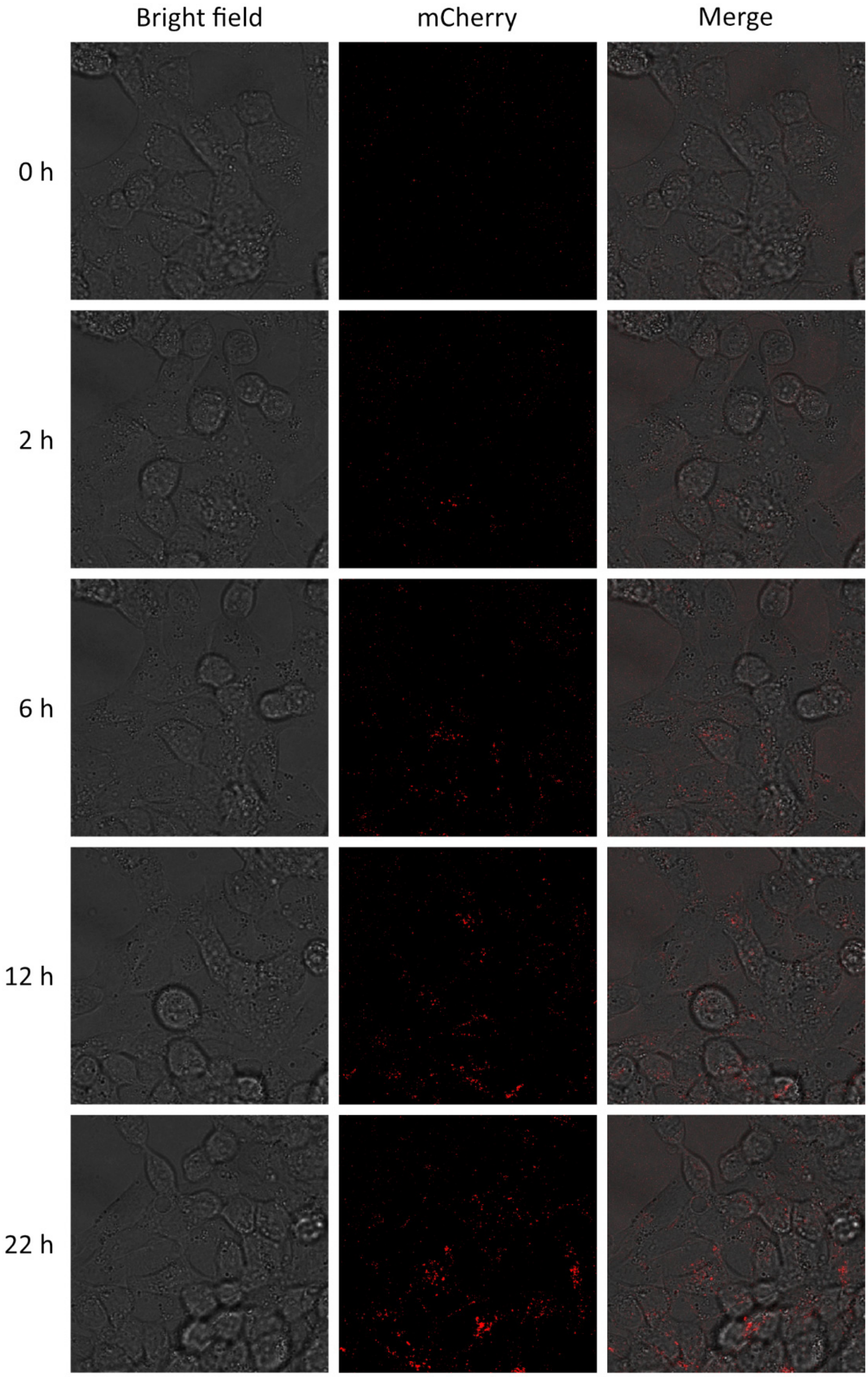

Supplementary Fig. S2 Fluorescence microscopy images of RL95-2 cells treated with the C5M1A at 100 nM with 100 nM of PD-L1-mCherry fusion protein over 22 hours.

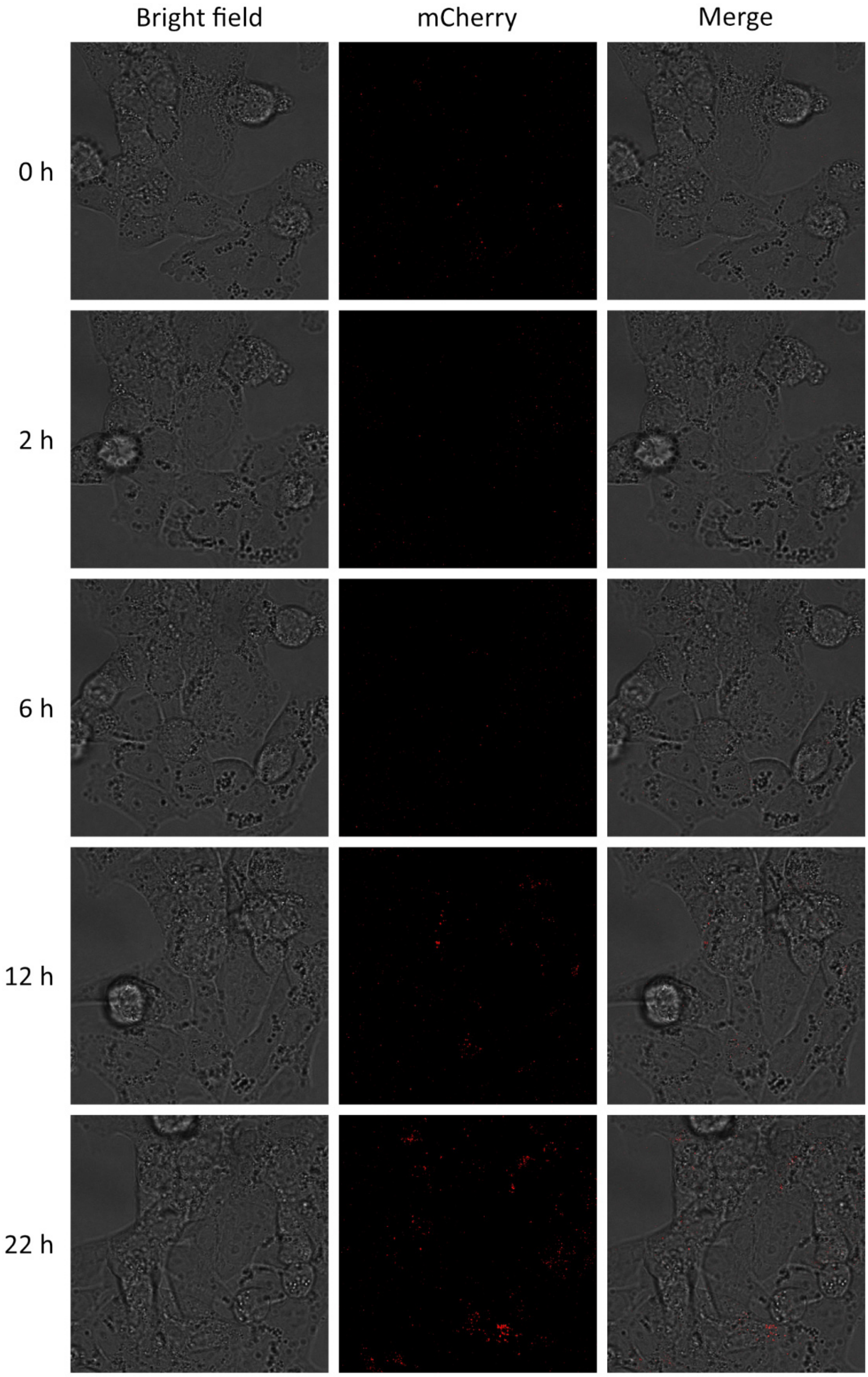

Supplementary Fig. S3 Fluorescence microscopy images of RL95-2 cells treated with the C5M1B at 100 nM with 100 nM of PD-L1-mCherry fusion protein over 22 hours.

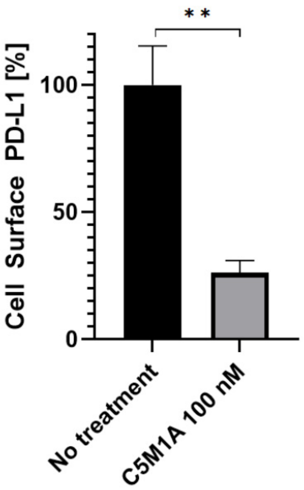

Supplementary Fig. S4 Determination of cell surface PD-L1 levels by live cell flow cytometry of RL95-2 cells treated with C5M1A. Data represent mean from 3 independent replicates after background signal substraction as mean ± SD. Untreated control was considered baseline level (100%). The unpaired t-test was used to compare mean of experimental group against the untreated control. P value threshold of less than or equal to 0.05 was considered statistically significant. ns: p > 0.05; * p ≤ 0.05; ** p ≤ 0.01; *** p ≤ 0.001; **** p ≤ 0.0001.

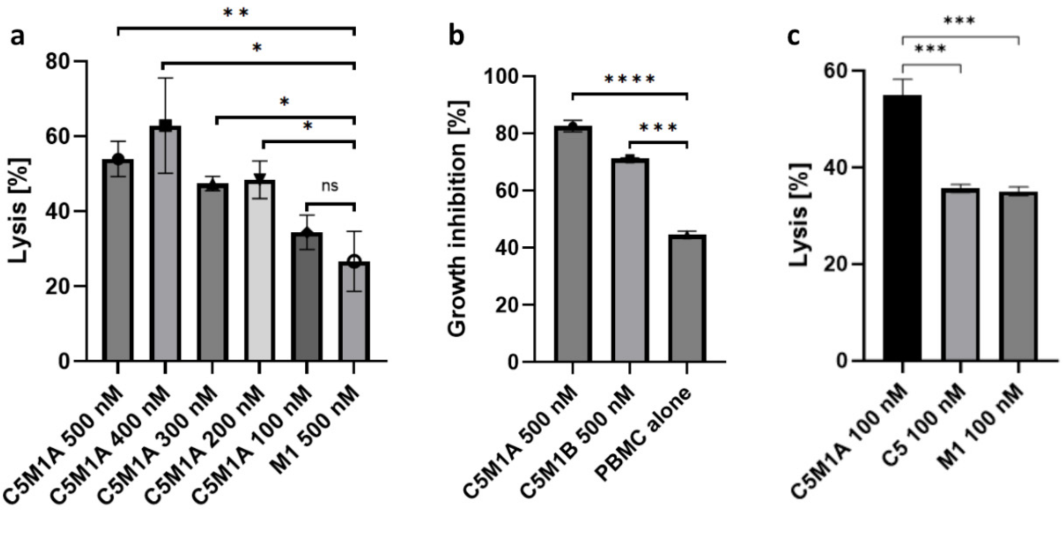

Supplementary Fig. S5 Results of PBMC cytotoxicity tests. **(a)** RL95-2 cells treated with C5M1A tested with PBMC from a single donor, compared to 500 nM M1-IgG1Fc chimera; **(b)** BT20 cells treated with C5M1A and C5M1B. **(c)** RL95-2 cells treated with C5M1A and control compounds: M1-IgG1Fc chimera and full C5 antibody. Data on all charts represent mean from 3 independent replicates as mean ± SD. The unpaired t-test was used to compare the means of each group against untreated control. P value threshold of less than or equal to 0.05 was considered statistically significant. ns: p > 0.05; * p ≤ 0.05; ** p ≤ 0.01; *** p ≤ 0.001; **** p ≤ 0.0001.

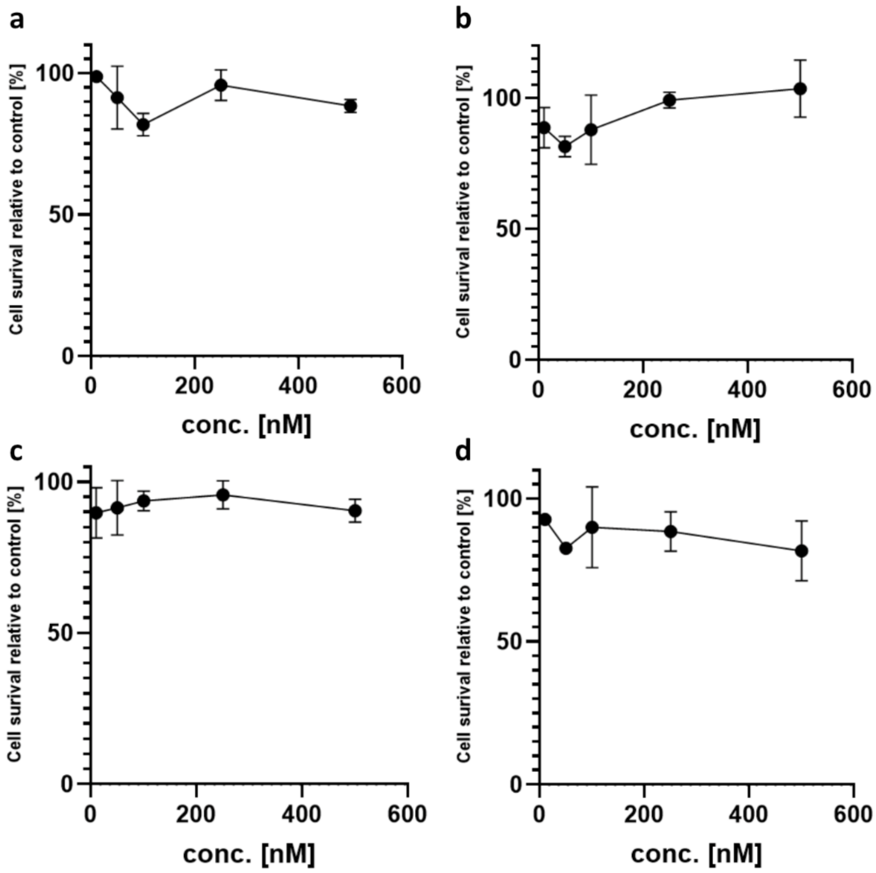

Supplementary Fig. S6 Cytotoxicity test of C5M1A. All used cell lines were analyzed: **(a)** RL95-2 **(b)** Panc 10.05 **(c)** PANC-1 **(d)** BT20
